# Going fishing: how to get what you want from a fungal genetic screen

**DOI:** 10.1128/msphere.00638-23

**Published:** 2024-07-03

**Authors:** Teresa R. O'Meara

**Affiliations:** 1 Department of Microbiology and Immunology, University of Michigan, Ann Arbor, Michigan, USA; University of Georgia, Athens, Georgia, USA

## Abstract

Five years ago, as I was starting my lab, I wrote about two functional genomic screens in fungi that had inspired me (mSphere 4:e00299-19, https://doi.org/10.1128/mSphere.00299-19). Now, I want to discuss some of the principles and questions that I ask myself and my students as we embark on our own screens. A good screen, whether it is a genetic or chemical screen, can be the starting point for new discovery and an excellent basis for the beginning of a scientific research project. However, screens are often criticized for being “fishing expeditions.” To stretch this metaphor to the extreme, this is because people are worried that we do not know how to fish, that we will come home without any fish, bring home the wrong fish, or not know what to do with a fish if we caught it. How you set up the screen and analyze the results determines whether the screen will be useful. In this mini-review, and in the spirit of teaching a scientist to fish, I will discuss recent excellent fungal genetic and chemical screens that illustrate some of the key aspects of a successful screen.

## WHAT IS THE BIOLOGICAL QUESTION?

This is probably the most critical aspect of starting a screen, and this was the highlight of my previous minireview (1). What is the phenotype, and how do you know that it is important? This step is going to require the most literature analysis and critical thinking, but without this, it is easy to chase down a phenomenon that will not significantly change our understanding. For a given organism, the important phenotypes are going to differ widely and of course will be a topic of hot debate.

For *Candida auris,* an emerging fungal pathogen, one of the major drivers for its outbreaks is the ability of a strain to adhere and form persistent biofilms on skin and medical devices. Therefore, when we started to investigate *C. auris,* our focus was to identify mutants that were unable to adhere to surfaces (2). Another major focus was on mutants that showed altered morphology, as multiple aggregating strains have been isolated from patients (3–5); screening for altered morphology was the focus of two recent insertional mutagenesis screens (6, 7). In other fungi, the interesting biological phenomena and topics of screens range from identifying regulators of sex and meiosis (8), defining essential genes (9–11), identifying kinases that regulate morphogenesis (12), determining modulators of host immune responses (13–15), or identifying new potential avenues for antifungal therapies (16–19), to name a few. The critical task is to define what the question is and to spend the time to justify why this is the right question to ask using a screening approach.

Importantly, not all fungi are compatible with all types of screens. The genetic toolbox that made *Saccharomyces cerevisiae* such a powerhouse in understanding biology is not available for all organisms, and developing these tools is a critical process in moving the field forward (20). Forward genetic screening starts with generating a collection of random mutants, screening for a phenotype, and then mapping the causative mutation. A classic approach is to use a mutagenic agent, such as ultraviolet (UV) or ethyl methanesulfate (EMS), to cause random mutations throughout the genome. A limitation of this approach is that these mutagens cause multiple mutations throughout the genome. For fungi that do not easily undergo sexual recombination, such as many of the fungal pathogens, identifying the causative mutant through back-crossing is not a viable option. Therefore, I will focus primarily on insertional mutagenesis screens. However, not all insertional mutagenesis approaches work with all fungi, and some of the transposon systems require significant engineering. Additionally, for diploid organisms, the transposons may only reveal haploinsufficiencies. As an alternate, it is possible to perform functional genomic screening, which is when a library of defined mutants is screened for a given phenotype. However, this requires a library of defined mutants, and these are not available for many fungi. The generation of these mutant collections is an ongoing and important advancement in the field.

Additionally, there should be some consideration about whether there are feasible secondary screens that will help validate the phenotype (more on this in Section 4). The advent of CRISPR-based tools to make reverse genetics possible has meant that follow-up assays are becoming more tractable, but the ease of genetic manipulation in the system still needs to be a factor in designing the screen.

## ASSAY DEVELOPMENT

General principles: Once the phenotype has been identified, the next step is to determine whether it is compatible with high-throughput genetic screening.

First, it needs to be scalable and quantitative. Measuring the phenotype should not be so labor-intensive that it would be impossible to perform it for many samples. Qualitative screens are possible, but developing quantitative measures will drastically increase throughput and decrease potential bias from the researchers. Pilot screens and small-scale analyses are enormously helpful for identifying the failure points of a new screen. Many assays that are tractable as individual experiments do not scale well or end up having more variability than anticipated when put into a high-throughput format. The advent of high-throughput automated microscopy means that imaging is now a fantastic tool for screening, as long as the analysis can be equally high throughput and quantitative. Imaging-based screening also allows for analysis of more complicated phenotypes than growth-based screening, including assaying for morphological changes (17) or differential phagocytosis by host cells (15).

Second, there should be both positive and negative controls. These controls are needed for determining the signal-to-noise ratio in the assay and for calculating the Z’ statistic. The Z′ is a measure of the dynamic range of the assay and indicates likelihood of false positives or negatives; the goal is to have a Z’ between 0.5 and 1 (21). The formula to calculate Z’ is $Z^{\prime }=\;\frac{3\left(\sigma_{p}+\;\sigma_{n}\right)}{\left|\mu_{p}-\mu_{n}\right|}$, where σ is the standard deviation, μ is the mean, and *p* and *n* are the positive and negative controls, respectively.

The variability of the wild-type should be considered for both technical and biological replicates. If the variability of the wild-type phenotype means that it cannot be distinguished from the positive control mutant, it will be near impossible to identify whether there is a hit. Even if the final screen will be performed in a single replicate, it helps perform the pilot screen with multiple replicates. The positive and negative controls are also important for understanding plate-to-plate or batch-to-batch variability, meaning each plate or batch should have both positive and negative controls.

As an example of a screen that would not work, I was interested in understanding the host factors that contribute to pyroptotic cell death in macrophages in response to *C. albicans* infection. Pyroptosis is an inflammatory cell death program that is driven by formation of an inflammasome comprising the NLRP3 protein, ASC, and caspase-1 in response to *C. albicans*. Mice that lack these components of the host immune response demonstrate decreased immunopathology during a systemic *C. albicans* infection (22). However, not every host cell gets infected with *C. albicans,* and of the infected cells, only approximately 10% of the cells form ASC +punctae (23). This means that the dynamic range of the assay when looking for loss of pyroptotic death is less than 10%. Most pooled host CRISPR screens have greater than 10% noise due to variability in gene targeting and biases in sequencing, so it would not be possible to get true hits from this screening design. Individual mutants could be assessed, but scaling it up to a genome scale was not possible.

**Fig 1:**
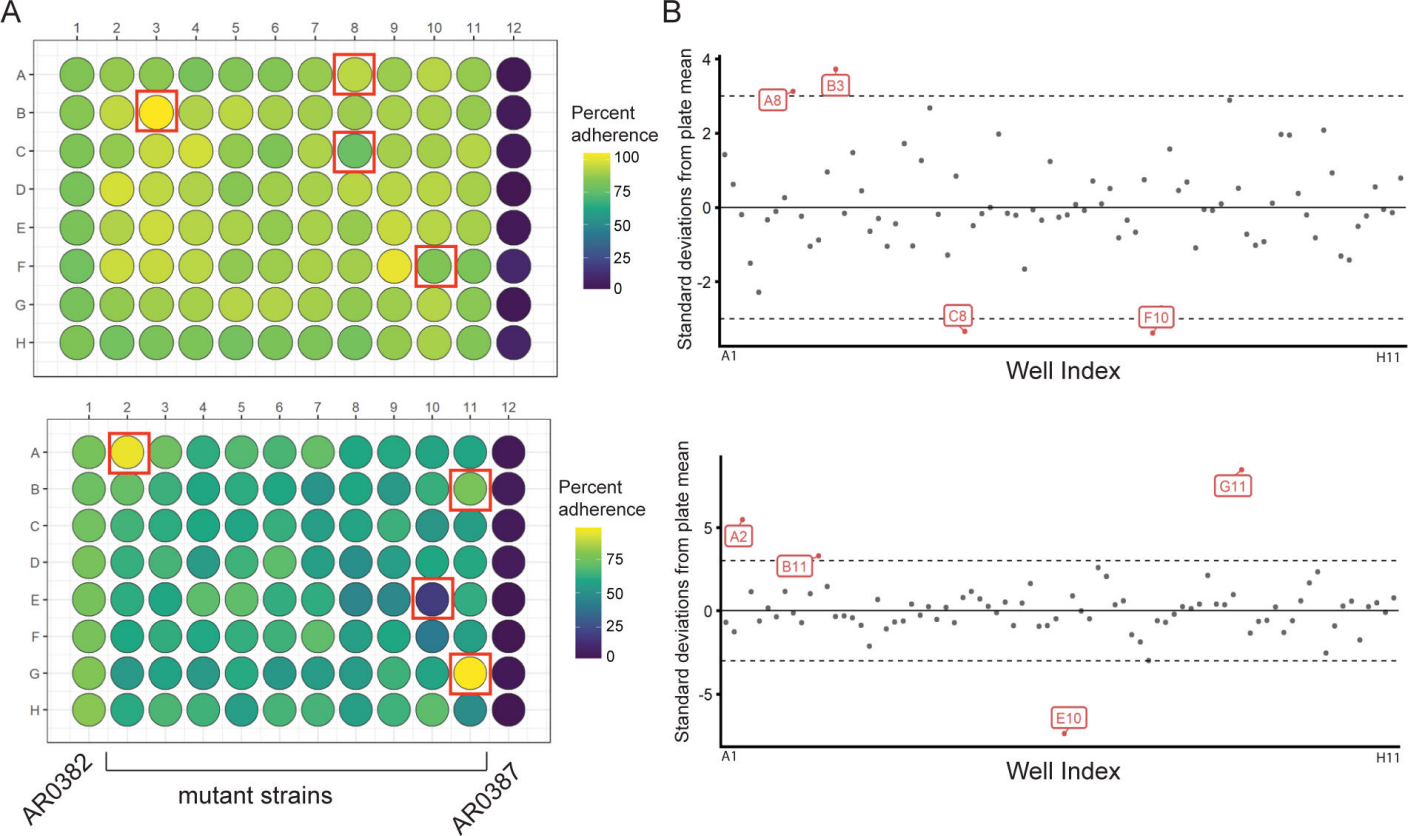
Screening for adherence regulators in *C. auris*. (**A**) Representative schematic of a 96-well plate layout for screening for adherence. In columns 1 and 12 are the negative (AR0382) and positive (AR0387) strains, respectively, with eight technical replicates each. Individual mutant strains generated by *Agrobacterium-*mediated insertional mutagenesis fill every other well (columns 2–11) in a single replicate. Colors indicate percent adherence of the cells in each well. The top plate represents a plate with overall high adherence, and the bottom plate has overall low adherence; this demonstrates the plate-to-plate variability that can occur during screens, even when controls are consistently separated. (**B**) The standard deviation from the plate mean for each mutant strain is plotted, and strains that are at least three standard deviations away from the mean were called as hits. For both A and B, strains that are called as hits in the initial screen are highlighted in red.

In contrast, when we were screening for adherence regulators in *C. auris,* we started with two clinical strains that differed significantly in their ability to adhere—AR0382 and AR0387. This meant that it was easy to identify strains with a strong defect in adherence (Fig. 1A). Even though there was variability from plate to plate, having two clinical isolates as controls as for both high and low adherence meant that we could normalize the data for each plate. In our case, we used a cutoff of three standard deviations from the mean per plate for calling a hit, and as shown in Fig. 1B, the cutoffs for calling a hit varied between each plate.

There are also screens where a sophisticated normalization scheme is needed to handle more complicated biases, such as cases where there are both plate and well-level effects. As an example, the density of host cells can impact their inflammatory capacity due to paracrine effects. Imaging-based analyses of inflammation would then need to account for cell density, which will vary across both the well and plate, and it may not be a linear response. To account for these biases, there are many nice reviews on the topic, which were generally developed for high-throughput chemical screens but can be adopted for fungal genetic screens as well (24–27).

Third, what is the cutoff for a hit? This combines aspects of the other points, but

“We’ll know it when we see it” is not the goal. What to consider a hit will encompass not only the statistical analysis of the data but also should take into consideration the tolerance for false-positive and false-negative results and the sensitivity of the assay. If the validation and follow-up assays are easy and fast, then the tolerance for false positives increases, and the set point for a hit can be more relaxed. In some cases, it is possible to use spike-in samples to determine the limit of detection in a pooled assay. This can be done by mixing different ratios of the positive and negative controls in a well before performing the screening assay and determining whether the analysis pipeline can detect the controls. Additionally, beyond the positive controls used to set up the assay, there are usually several expected hits identified in the screen; these are genes or drugs that have known function and can serve as a sanity check that the screen is working and that the thresholds are set at the appropriate sensitivity.

## MAPPING OF HITS

### Forward genetic screening

During forward genetic screening, where unknown mutants are assayed for their behavior, identifying the causative mutation is challenging. For insertional mutagenesis assays, the classical approach is to use inverse PCR to map the insertion site (6). An interesting test case where inverse PCR was used to identify the target of interest was in the identification of an lncRNA that negatively regulated elongated cell morphology in *C. auris* (6). The transposon insertion event led to a wrinkly colony morphology, which was visible on the plate. Through inverse PCR, the authors identified that the insert occurred between two genes. However, the authors realized that transcription was occurring from this region and identified a *C. auris-*specific lncRNA that they named DINOR, which was responsible for the phenotype (6). Moreover, they were able to delete this lncRNA in multiple clades of *C. auris* and observed the same elongated cell phenotype in the mutants, demonstrating conservation across *C. auris*.

It is also possible to take advantage of decreasing costs in whole-genome sequencing to perform pooled mapping of a small set of strains (7, 28). Again, this is compatible with arrayed or otherwise separated strains but would be difficult in large-scale pools due to the need for extreme sequencing depth. In the small-scale pools, rather than mapping the genome sequencing reads to the genome, the reads are mapped to the insert sequence. Any reads that partially map to the insert are those that cover the insert boundaries; these can be collected and mapped to identify the insertion sites (7, 28). Then, through PCR, it is possible to deconvolute the pools to determine which insert goes with which strain. This can be cost-effective and easier than inverse PCR. Using this approach, we were able to rapidly map five *Agrobacterium-*mediated t-DNA inserts that resulted in wrinkly colony morphology in *C. auris* (7). However, most of the genome sequencing reads that are generated are not informative, as they cover the rest of the uninterrupted genome, thus limiting the utility of this approach for a large pool of strains.

TnSeq represents the next generation of insertion mapping, where the insertion sites are mapped in a large, pooled library of mutants that each contain a transposon. Generally, TnSeq libraries are created at saturating mutagenesis levels, allowing for whole-genome analysis of a particular phenotype. PCR-based amplification of the insert site coupled with high-throughput sequencing allows the insert sites to be mapped, and by comparing the frequency of insertions pre- and post-phenotyping screening, it is possible to identify mutants that are over or underrepresented in the pool. Essentiality gene mapping is a special case, as only non-essential genes will be recovered in the initial pool. In this study, the absence of strains with insertions into a particular gene will indicate that a gene is essential. However, it is also possible that insertions into specific regions of a gene will allow for maintenance of viability where a full gene deletion would not—this can help identify domain-specific information about viability. Recent work in *Candida glabrata* used the Hermes transposon system to map essential genes (29) and found that only 84% of the essential genes in *C. glabrata* were also essential in *Saccharomyces cerevisiae* (29). In *C. albicans,* which is normally diploid, Segal and colleagues used an engineered haploid strain to perform TnSeq with the AcDs transposon system and identify essential genes (9). Similarly, the essential gene list in *C. albicans* differed from both *C. glabrata* and *S. cerevisiae* (9).

### Functional genomic screening

A distinct advantage of functional genomic screening is that the mutant strains are already known; therefore, the gene identity of the hit is straightforward. A limitation of forward genetic screening is the time-consuming nature of preparing a library of defined mutants, which means that mutant collections are generally only made in a single-strain background. Recent work on strain variation in fungi has demonstrated significant differences in phenotypes between the same deletion or mutation in different strain backgrounds (30–33). As described above for the lncRNA *DINOR* (6)*,* important validation steps can include making the deletion in multiple strain or clade backgrounds to determine the conservation of the phenotype.

### Special cases: Genome-wide association studies and natural variants

Genome-wide association studies are a powerful tool for identifying the genetic basis of variation in phenotypes of interest. Rather than screening a collection of mutants, it is possible to screen a collection of natural isolates. This approach leverages the natural variation that occurs in species and allows for both gain-of-function and loss-of-function phenotypes. Application of GWAS approaches in bacteria has led to the identification of underlying traits of genetic variation, such as antibiotic resistance, invasiveness, and preference in site of colonization and infection (34, 35). In human fungal pathogens, an analysis of the *A. fumigatus* pan-genome identified variants that were associated with increased virulence and drug resistance (11). Genome analysis and phenotypic profiling of a population of *Clavispora lusitaniae* isolates obtained from a cystic fibrosis lung identified a specific set of mutations in Mrr1 critical for conferring resistance to fluconazole (36). In non-sexual species, it can be difficult to use GWAS as many loci are linked through common descent because of the lack of meiotic recombination. The large amount of genetic diversity between isolates is thus a hindrance to identifying the causative differences. An alternate approach is to use multiple isolates from the same source with a limited amount of sequence divergence, but a difference in phenotypes. By using this approach in *C. albicans,* we were able to identify a specific dominant negative allele in a transcription factor, Zms1, with a role in invasive growth (32). Similarly, an analysis of related *C. albicans* isolates from a single cystic fibrosis patient lungs identified a gain-of-function allele in the Rob1 transcription factor, which also drove invasive growth (37).

## VALIDATION

After the high-throughput screening and the identification of hits, it is important to perform validation assays. When screening a mutant library, validation should include generating individual independent knockouts and complementation strains. These independent knockouts and complementation strains are necessary to demonstrate that the phenotype is due to the specific mutation and not from a spurious event that occurred during the mutagenesis process. For example, it is possible that the DNA breaks that occur during mutation lead to chromosomal fusion events, or a loss of heterozygosity, or other random mutations. These can have large consequences on phenotypes, so the reintroducing of the wild-type allele and complementing the phenotype back to wild-type is critical for determining the genotype-to-phenotype connection.

After generation of independent mutant lines, this is the time for a good secondary validation assay. These are usually done in low throughput and ideally would be an orthogonal measure of the screen. To validate our screen of adherence regulators in *C. auris*, we used binding to fluorescent polystyrene microspheres and flow cytometry, which both increased the dynamic range of the assay and showed the generalizability of the phenotype across surfaces (2). For the TnSeq screen to identify sexual regulators in *S. pombe,* Billmyre and colleagues turned to a viable spore yield assay as a secondary measure of fertility, as well as individual microscopy of spore structure and morphology (8). These tests were important as they helped define the specific defect of the *plb1∆* and *alg1∆* mutant strains in gamete health rather than initial formation of spores.

## PRIORITIZATION AND FOLLOW-UP

Most screens will give many hits that pass the significance thresholds and will be validated in a secondary analysis. The question then becomes how to focus on the most interesting hits. In this study, the focus is on understanding what classes of genes might emerge from the screen and determining what alternate explanations might exist for the hits. It is possible to get hits that are due to the nature of the screening assay. For example, if the screening readout is increased reporter fluorescence, the hit may be something involved in protein degradation, which can change fluorescence but not be related to the phenotype of interest. It is worthwhile to spend time thinking of alternative explanations for the data and designing secondary assays to rule out these unrelated hits before spending a lot of time and effort chasing down mechanism.

As a nice example of this, Billmyre and colleagues walk through multiple alternate explanations for their hits, including insertion io essential genes with long-lived transcripts that had not fully been eliminated during mutant pool generation or those with non-sexual-related growth defects (8). By rapidly ruling out these hits, they were able to focus specifically on those with interesting biology and a specific defect in sexual reproduction. In another nice example, Rahman and colleagues screened a library of transcription factor deletion strains in *Aspergillus fumigatus* for altered interactions with epithelial cells (13). The lung epithelium is usually the first line of host contact with germinating *Aspergillus,* and so understanding this specific host–pathogen interaction is critical for understanding *A. fumigatus* infections. After identifying hits, they performed secondary screens for germination rate, hyphal extension rate, phagocytosis and adhesion, and growth in different stresses. These secondary assays can help bin mutants into different classes, which can then help focus the downstream analysis onto the most interesting genes.

What happens if the most interesting hit is in a gene of unknown function? This means there is a phenotype for the knockout, but you are missing biochemical understanding of how a particular gene contributes to that function. In non-model systems like many fungal pathogens, this is still very common. New computational tools can help predict a function, taking advantage of advancements in sequencing and structure-based analysis. These include coexpression analysis (38–41) for a guilt-by-association approach, or structural comparisons using tools like FoldSeek (42) or LazyAF for predicting protein–protein interactions (43) that can help pull in information from better characterized proteins. Using these computational predictors can help suggest a specific function that can then be more carefully tested using the appropriate biochemical or molecular assays. As these molecular assays are generally more laborious than the computational approaches, using multiple lines of evidence will be important so that you can design the right experiment rather than having to test many different potential functions.

## RECENT INNOVATIVE SCREENS

I will discuss recent fungal genetic screens that focused on complicated phenotypes to demonstrate how a well-designed screen can give new insights into biological processes.

*C. albicans* morphogenesis is an important virulence trait, but the cues and signaling cascades that govern morphogenesis are still being determined. Wakade and colleagues developed an intravital imaging model that allowed for a detailed analysis of morphogenesis during infection (44). They started with the Homann library of 155 transcription factor mutants produced in the SC5314 lineage of *C. albicans* and added a p*ENO1-RFP* fluorescent reporter into each strain to allow for fluorescent imaging. By competing with a p*ENO1-GFP* marked parental strain, they could compare the ratio of filamentous cells to the parent, thus allowing for normalization and a positive control within each experiment. They identified 19 mutants with a significant reduction in filaments compared with the reference strain and were able to categorize the strains as either core or auxiliary filamentation regulators (44). They confirmed the phenotype of the *efg1∆/∆* mutant by leveraging a clinical strain, P94015, with a natural variant of Efg1, showing the generalizability of the phenotype across backgrounds. Another of their hits, the *stp2∆/∆* mutant, was found to be due to a genetic interaction with *arg4∆/∆* mutation used to generate the strain, and by adding *ARG4,* we were able to revert the phenotype. This is a nice example of how the authors were able to rule out a specific mutant for being unrelated to the process of interest.

During gut colonization, *C. albicans* is decorated by immunoglobulin A (IgA), which actually increases the competitive fitness of the fungus (45). To determine which fungal proteins are recognized by IgA and thus determine new processes involved in gut colonization, Ost and colleagues screened a library of *C. albicans* deletion strains for alterations in IgA binding. They used flow cytometry to measure the levels of bound IgA per fungal cell. Importantly, this screen was performed on IgA collected from both Swiss Webster and C57BL/6 mice, which demonstrated the generalizability of the binding. This led to the identification of Ahr1 as a critical transcription factor, as it was a hit from both mutant collections and in response to both IgA sources. As Ahr1 regulates morphogenesis, they used the constitutively filamentous TetOn-NRG1 to show that hyphae are more bound by IgA and, moreover, that a hyphal-specific adhesin, Als3, is the main target of intestinal IgA binding (45). To extend the results, they also demonstrated *Candida glabrata* is targeted by IgA, using *S. cerevisiae* strains that express specific *C. glabrata* adhesin proteins. This is an example of how a screen for a specific host response can lead to new insights into the complicated interactions between hosts and fungi.

To understand the *Cryptococcus neoformans* signaling cascades that contribute to virulence, Bahn and colleagues undertook a systematic functional genomic screening approach and generated individual barcoded deletions of all non-essential phosphatases, kinases, and transcription factors in the *C. neoformans* genome (46, 47). The major focus was on mutants with altered virulence in two models: mouse and insect. Secondary assays included comprehensive *in vitro* growth profiling, which revealed that many of the mutants with pathogenesis phenotypes also had a defect in least one growth condition. Capsule and melanin, which are two major virulence traits of *C. neoformans,* were likewise often impacted by mutation in these signaling cascades (46, 47). By performing both *in vitro* and *in vivo* analyses of the mutant strains, this screening approach was able to define *in vitro* phenotypes that were best correlated with disease progression and fungal burden *in vivo*.

Beyond fungal growth, disease is also determined by host immune responses. *C. neoformans* infections are characterized by a strong Th2 response, rather than a pro-inflammatory Th1 response. Dang and colleagues screened a collection of 4,402 individual deletion strains in *C. neoformans* to identify strains that were unable to drive macrophage M2 polarization by measuring induction of arginase-1 using flow cytometric analysis of infected bone marrow-derived macrophages (14). They re-screened their top 100 hits and were able to validate 14 strains with a significant defect in arginase-1 production. As an orthogonal assay, they demonstrated that their top hit, Cpl1, was important in preventing iNOS expression induced by the inflammatory stimuli LPS and IFNγ (14) and that heterologous expression of Cpl1 was sufficient to induce arginase-1. Through these screens, they were able to identify a fungal secreted effector that enhances type 2 inflammation and disease.

Two recent TnSeq screens in *Candida glabrata* have focused on fluconazole resistance and susceptibility (29, 48), as a major challenge in treating *C. glabrata* is its intrinsic drug resistance. For each of these screens, the authors looked for insertional mutants that were differentially represented in the pool after exposure to fluconazole. Gale and colleagues used a treatment that included 24 hours of fluconazole admiistration, followed by 24 hours of no drug media—this allowed them to assay for genes that not only impacted survival in drugs but also those genes that impacted cellular recovery after stress (29). Chow and colleagues used three doses of fluconazole for 24 hours (48). As expected, each screen found the drug efflux pumps as hits and the UPC2 transcription factor as hypersensitive, confirming the results of screening approach. Moreover, in both screens, mitochondrial mutants that were resistant to fluconazole were identified, though each group followed up on a different set of mutants in this class. To validate the high-throughput analyses, the authors generated clean deletions of each candidate hit and performed minimum inhibitory concentration assays to determine the degree of sensitivity or resistance to fluconazole. From these approaches, it was possible to identify new targets for combination therapies with fluconazole.

## CONCLUSIONS AND OUTLOOK

**Fig 2:**
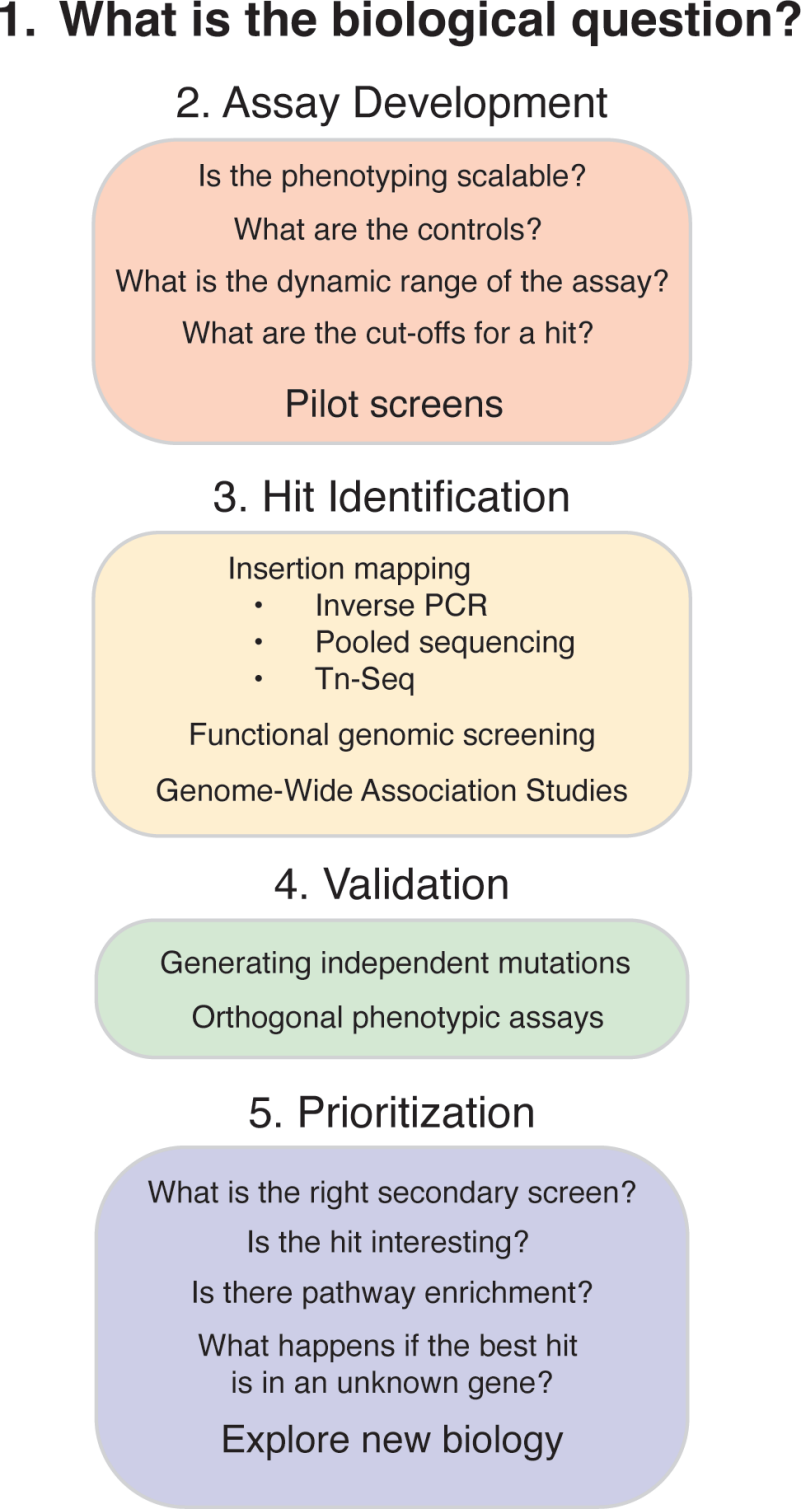
Questions to ask when starting a screen. In each colored box are the sub-questions and goals that should be addressed for each of the steps.

Genetic screens can allow you to explore uncharted waters and find new genes involved in exciting biological processes. Going through the steps and questions in Fig. 2 can help with the logic of designing a fruitful screen. As new genetic tools become available for more organisms, including CRISPR mutagenesis as well as CRISPRi and CRISPRa tools (7, 49–52), the ability to perform genetic screening is vastly increasing. An exciting new direction is the ability to perform genetic screening in multiple strain backgrounds, which allows for an exploration of the consequences of genetic background and environment on a phenotype. Very few traits are monogenic, and understanding how genes influence each other is going to be fundamental in understanding the genotype-to-phenotype question that underlies genetics research. The limitation in this study is to find the right phenotype to explore using a well-designed genetic screen.
